# Supplementary figures and images for: Isolation of soil bacteria able to degrade the anthelminthic compound albendazole
Source: PeerJ. 2023 Nov 6;11:e16127. doi: 10.7717/peerj.16127 (PMC10634332; doi:10.7717/peerj.16127)

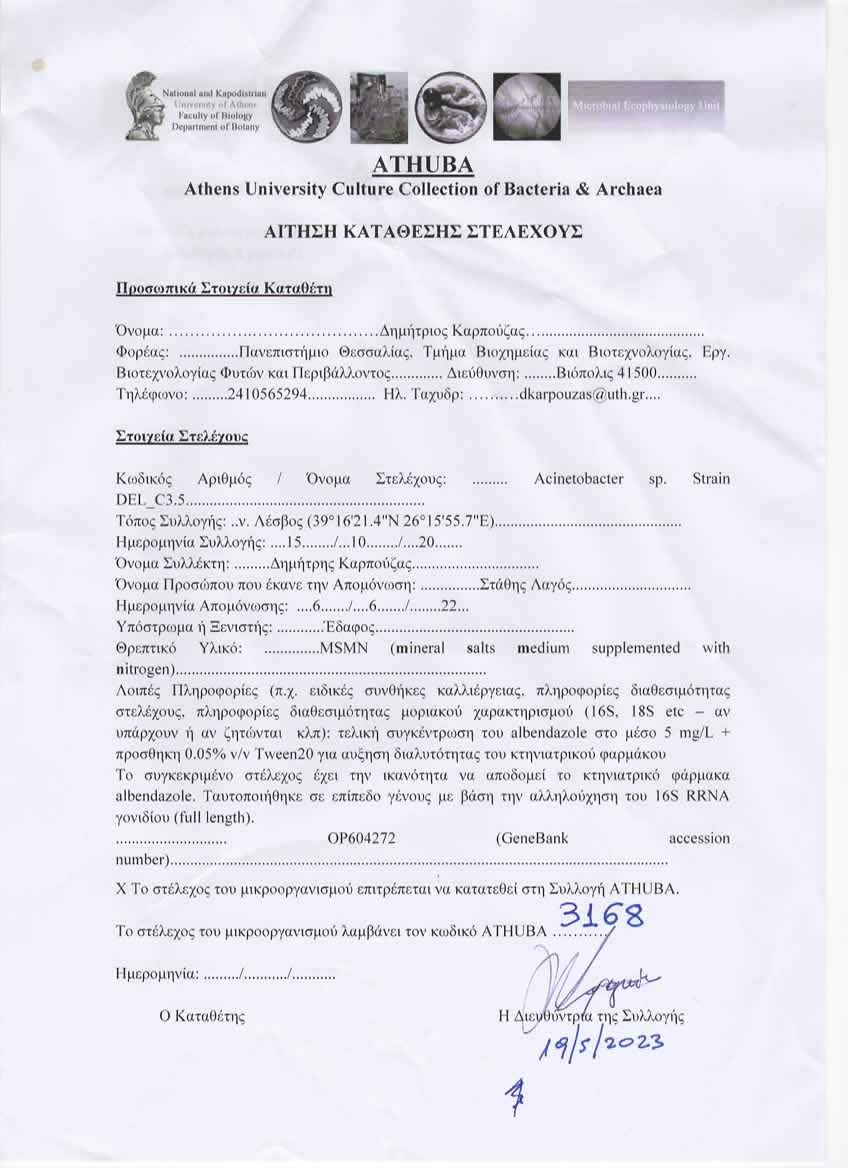

Supplement: Supplemental Information 4 [file peerj-11-16127-s004.jpg]

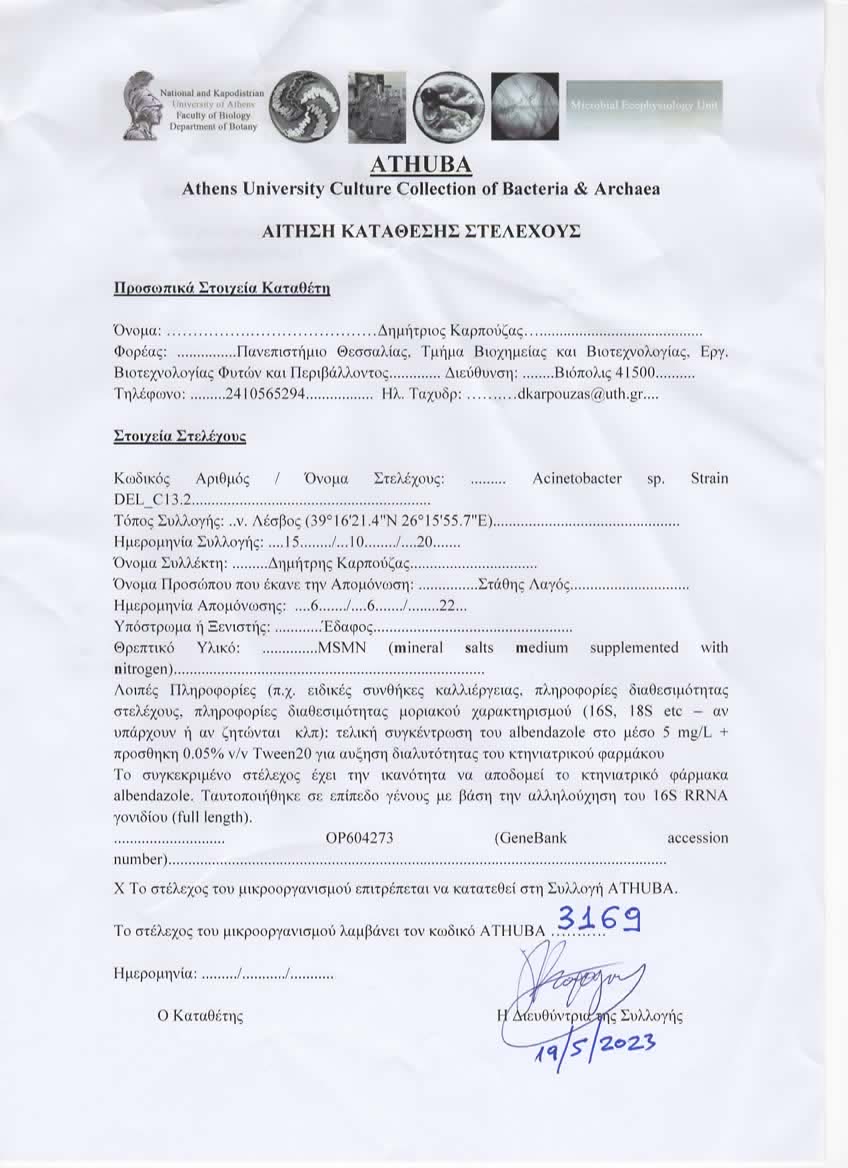

Supplement: Supplemental Information 5 [file peerj-11-16127-s005.jpg]
